# Scaling behavior of nanoimprint and nanoprinting lithography for producing nanostructures of molybdenum disulfide

**DOI:** 10.1038/micronano.2017.53

**Published:** 2017-09-11

**Authors:** Mikai Chen, Hossein Rokni, Wei Lu, Xiaogan Liang

**Affiliations:** 1 Mechanical Engineering Department, University of Michigan, Ann Arbor, MI 48109, USA

**Keywords:** 2D materials, charge trapping, memory, MoS_2_, nanoimprint, nanoprint

## Abstract

Top-down lithography techniques are needed for manufacturing uniform device structures based on emerging 2D-layered materials. Mechanical exfoliation approaches based on nanoimprint and nanoprint principles are capable of producing ordered arrays of multilayer transition metal dichalcogenide microstructures with a high uniformity of feature dimensions. In this study, we present a study on the applicability of nanoimprint-assisted shear exfoliation for generating ultrathin monolayer and few-layer MoS_2_ structures as well as the critical limits of feature dimensions produced via such nanoimprint and nanoprint-based processes. In particular, this work shows that give a lateral feature size of MoS_2_ structures that are pre-patterned on a bulk stamp, there exists a critical thickness or aspect ratio value, below which the exfoliated layered structures exhibit major defects. To exfoliate a high-quality, uniform monolayer or few-layer structures, the characteristic lateral feature sizes of such structures need to be in the sub-100 nm regimes. In addition, the exfoliated MoS_2_ flakes of critical thicknesses exhibit prominent interlayer twisting features on their cleaved surfaces. Field-effect transistors made from these MoS_2_ flakes exhibit multiple (or quasi-analog-tunable) charge memory states. This work advances the knowledge regarding the limitations and application scope of nanoimprint and nanoprint processes in manufacturing nano/microstructures based on layered materials and provides a method for producing multi-bit charge memory devices.

## Introduction

Two dimensional (2D)-layered transition metal dichalcogenides (TMDCs) (for example, MoS_2_, WSe_2_, and WS_2_) have been widely studied as attractive material candidates for making electronic, electrical, photonic, biological, and chemical sensing devices with either significantly improved performance or a new functionality^1–9^. Despite the breakthroughs and progress in demonstrating TMDC-based prototype devices, the research and industry communities still lack scalable manufacturing techniques that are capable of producing orderly arranged, high-quality, highly uniform TMDC device structures over wafer-scale areas. In particular, the community needs top-down lithography manner techniques that are capable of producing pre-structured TMDC nano/microstructures with deterministic and uniform feature dimensions and physical properties. Our research team recently developed and investigated a nanoimprint/nanoprint-based method that is capable of producing multilayer MoS_2_ device structure arrays with a high device-to-device consistency in feature dimensions and electronic/biosensing characteristics^10^. This approach is called nanoimprint-assisted shear exfoliation (NASE). In our previous works, NASE processes were demonstrated to be capable of generating multilayer TMDC structures with characteristic lateral feature sizes in the range of 7 to 25 μm and vertical thicknesses in the range of 20–200 nm^10,11^. Additional effort is needed to identify the limits of the critical feature dimensions produced by NASE processes and further extend the application scope of NASE for producing ultrathin monolayer and few-layer TMDC device structures.

In this study, we present a study on the important limits of the feature dimensions of MoS_2_ nanostructures (the most widely studied TMDC structures) produced by shear exfoliation in combination with transfer printing. In particular, we experimentally evaluated the viability of nanoimprint and nanoprint processes for producing ultrathin monolayer and few-layer MoS_2_ structures. More specifically, our work shows that the quality of mechanically exfoliated MoS_2_ nano/microstructures highly depends on the aspect ratio (or the ratio between feature thickness (or height) and lateral feature size) of pre-patterned structures on a bulk MoS_2_ stamp. For exfoliated MoS_2_ features with a given lithographically defined characteristic lateral size, the vertical thicknesses (or aspect ratios) of these features have a critical limit, below which the exfoliated MoS_2_ features exhibit major defects that are caused by mechanical exfoliation processes. For example, for exfoliated MoS_2_ features with lateral feature sizes larger than 5 μm, the critical aspect ratio is estimated to be approximately 1:430; for exfoliated MoS_2_ nanostructures with lateral sizes smaller than 1 μm, the critical aspect ratio is approximately 1:47. This strongly implies that to generate high-quality ultrathin monolayer or few-layer MoS_2_ structures using nanoimprint and nanoprint approaches, the lateral dimension sizes of pre-patterned MoS_2_ features on a bulk stamp need to be set in the sub-100 nm regime. In addition, the exfoliated MoS_2_ structures, whose thicknesses or aspect ratios are close to corresponding critical values, exhibit prominent top-view Moiré patterns on the cleaved surfaces, indicating the existence of interlayer twisting features in the MoS_2_ layers close to these cleaved surfaces. Field-effect transistors made from such MoS_2_ structures exhibit multiple (or quasi-analog-tunable) charge memory states, which could be further exploited to make analog computing and multi-bit memory devices.

## Materials and methods

**Figure 1:**
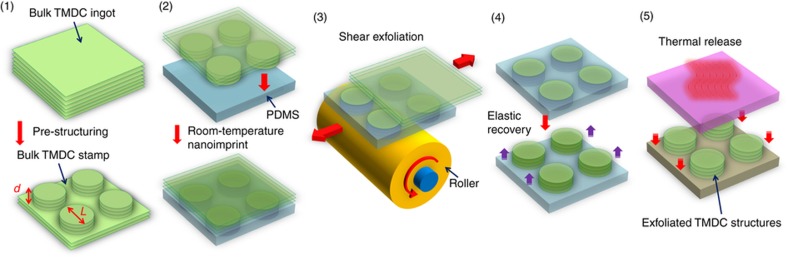
Schematic illustration of nanoimprint-assisted shear exfoliation and transfer-printing (NASE+TP) processes for generating pre-patterned nanostructures of layered materials.

**Figure 2:**
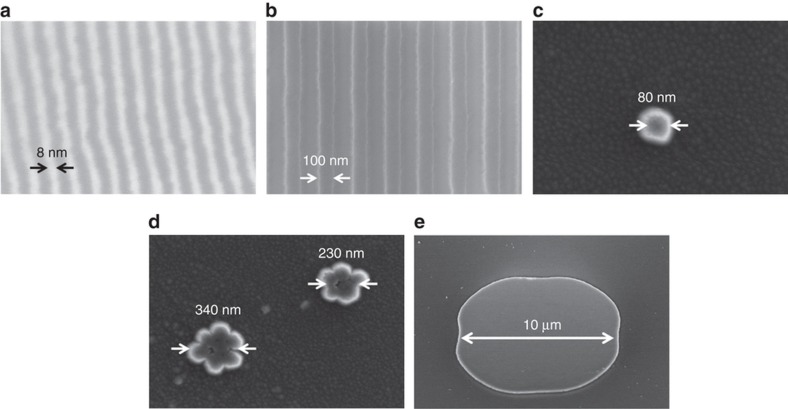
SEM images of a set of representative bulk MoS_2_ stamps bearing (**a**) 8-nm half-pitch line-spacing patterns, (**b**) 100-nm half-pitch line-spacing patterns, (**c**) 80-nm pillars, (**d**) hundreds of nm-sized mesas, and (**e**) 10-μm mesas.

Figure 1 schematically illustrates the nanofabrication method under study, which involves a NASE process followed by a transfer-printing (TP) step and is therefore referred to as the NASE+TP method. Specifically, this NASE+TP method includes (a) pre-fabrication of a bulk MoS_2_ stamp-bearing protrusive to-be-exfoliated device structures with deterministic lateral sizes and thicknesses, (b) room temperature nanoimprinting of protrusive to-be-exfoliated MoS_2_ features into a polydimethylsiloxane (PDMS) stamp, (c) shear exfoliation of imprinted MoS_2_ features into the PDMS stamp using a roller tool, (d) elastic recovery of the PDMS stamp, elevating the as-exfoliated MoS_2_ structures onto the flat PDMS surface, and (e) transfer printing of the MoS_2_ features onto the target substrate via a thermal release process. In this work, the bulk MoS_2_ ingots for making nanoimprint stamps are from SPI (SPI Supplies, West Chester, PA, USA) (sample size of ~1 cm^2^). The pre-patterning of the MoS_2_ stamps with protrusive microstructures (that is, the structures with microscale characteristic lateral feature sizes) was performed using a method previously reported by our group^12^. To pre-pattern nanostructures with various nanoscale lateral feature sizes on the stamps, we exploited a series of different lithography techniques, including block copolymer (BCP) self-assembly^13^, nanoimprint lithography (NIL)^12^, and nanoparticle (NP) lithography. Specifically, polystyrene-block-polydimethylsiloxane (PS-b-PDMS) copolymers were used for generating sub-10 nm size line-spacing patterns. The details of the BCP processes can be found in our previously published works^13–15^. NIL processes were performed to generate polymeric nanopatterns with characteristic lateral feature sizes in the range of 100 nm to 1 μm^12^. Furthermore, 80 nm Au NPs were randomly dispersed onto MoS_2_ ingot surfaces to form NP cluster patterns with characteristic lateral sizes ranging from 80 to 500 nm, which can serve as etching masks for generating protrusive MoS_2_ mesas with various lateral sizes (note that such randomly distributed NP/cluster patterns are not used to create any ordered device structures but purposely generate MoS_2_ mesas with a broad range of lateral feature sizes and therefore enable the study of the effects of different aspect ratios of to-be-exfoliated MoS_2_ nanostructures on the exfoliation results). With such BCPs, NIL-generated polymeric and NP/cluster nanopatterns as the etching masks, protrusive to-be-exfoliated nanostructures on the bulk MoS_2_ stamps were formed using SF_6_ reactive ion etching (RIE) (processing parameters: SF_6,_ flow rate: 20 sccm, chamber pressure: 20 mTorr, RF power: 200 W). The etching rate was ~100 nm min^−1^. Figure 2 displays the scanning electron micrographs (SEMs) of several representative MoS_2_ stamps bearing the protrusive nano/microstructures with various characteristic lateral feature sizes and vertical heights (or thicknesses), which include (a) 8-nm half-pitch line-spacing structures patterned from PS-b-PDMS BCP features, (b) 100-nm half-pitch line-spacing structures formed by NIL processes, (c) 80-nm and (d) hundreds of nm-sized protrusive mesas patterned from randomly distributed Au NPs and clusters, and (e) 10-μm protrusive mesas formed using regular photolithography followed by RIE. In summary, the fabricated MoS_2_ stamps bear to-be-exfoliated nano/microstructures with characteristic lateral feature sizes ranging from 8 nm to 50 μm and vertical feature heights (or thicknesses) ranging from 0.8 nm (monolayer structures) to 500 nm (~770 layers).

During the NASE+TP process, a lab-made motorized roller tool is used to firmly press the MoS_2_ stamp against the PDMS film (vertical gauge pressure of ~2×10^6^ Pa) and simultaneously generate a shear displacement (relative velocity 0.1–1 mm s^−1^) between the protrusive MoS_2_ structures mechanically imprinted into PDMS and the bulk stamp via the friction force between the rotating roller and the bulk stamp^10,11^. Because of this shear displacement, the imprinted MoS_2_ layers can be exfoliated away from the stamp along the shear direction^10,11^. After this shear exfoliation step, the vertical imprint pressure is released, and the surface of the elastic PDMS film quickly restores back to the flat morphology, forming a flat PDMS stamp that bears exfoliated MoS_2_ nano- and microstructures. During the NASE process, the thicknesses of the exfoliated MoS_2_ flakes are mainly determined by the imprinting depth (that is, the pre-defined height) of the protrusive MoS_2_ structures that are pre-patterned on the bulk MoS_2_ stamp^10,11^. During the transfer-printing step, the MoS_2_ nano- and microstructures on the PDMS stamp are mechanically pressed against the target Si substrate that is coated with 300-nm-thick thermally grown SiO_2_. Afterwards, the PDMS stamp is heated up to 200 °C using a heater, and the MoS_2_ nano- and microstructures are thermally released from the PDMS stamp and subsequently transferred onto the target Si/SiO_2_ substrate.

Scanning electron microscopy (SEM) images of as-exfoliated MoS_2_ nano- and microstructures were taken using an in-line field-emission SEM (Manufacturer: Hitachi High Technologies America, Schaumburg, IL, USA; Model Number: Hitachi SU8000 In-line FE-SEM) with an accelerating voltage of 2 kV. High-resolution transmission electron microscopy (HRTEM) images of the cleaved surfaces of the MoS_2_ structures were obtained using a JEOL 2010F analytical electron microscope with an accelerating voltage of 200 kV.

## Results

**Figure 3:**
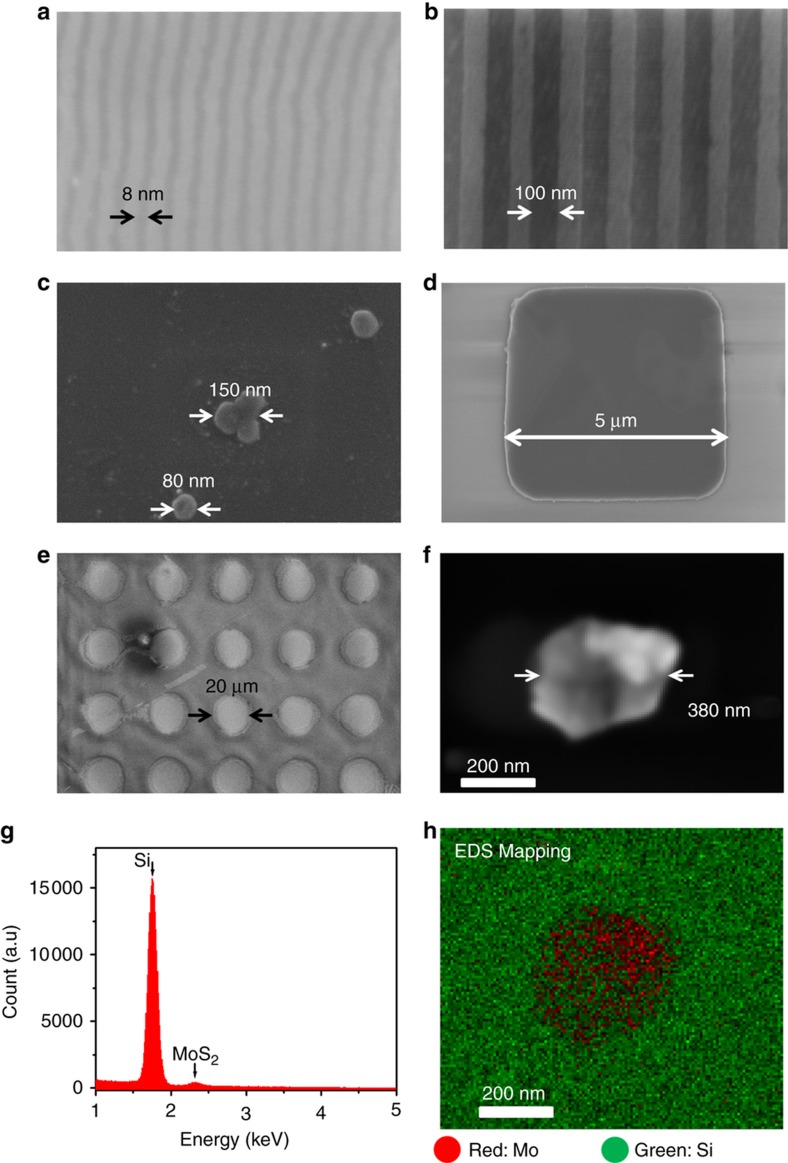
SEM images of representative good-quality MoS_2_ nano- and microstructures produced via NASE+TP processes, including (**a**) 8 nm half-pitch, 1.3 nm-thick line-spacing patterns, (**b**) 100 nm half-pitch, 3.3 nm-thick line-spacing patterns, (**c**) 80–150 nm size, 10 nm-thick mesas, (**d**) 5 μm size, 25 nm-thick flakes, (**e**) 20 μm size, 40 nm-thick flakes, and (**f**) a 380 nm size, 20 nm-thick flake. (**g**) The EDS spectrum acquired from the MoS_2_ nanostructure shown in (**f**). (**h**) The EDS elemental mapping of Mo and Si.

Figure 3 displays the SEM images of the representative MoS_2_ nano/microstructures produced by the NASE+TP processes performed under the same processing condition (vertical gauge pressure of ~2×10^6^ Pa, shear velocity of ~0.5 mm s^−1^), which have relatively large feature aspect ratios (that is, feature thickness (*d*)/characteristic lateral feature size (*L*)) and exhibit a high feature quality. These nano/microstructures include (a) an 8-nm half-pitch, 1.3-nm-thick line-spacing structures (aspect ratio of approximately 1:6), (b) a 100-nm half-pitch, 3.3-nm-thick line-spacing structures (aspect ratio of ~1:30), (c) 80–150-nm size, 10-nm-thick mesas (aspect ratios: 1:8–15), (d) 5-μm size, 25-nm-thick flakes (aspect ratio of ~1:200), (e) 20-μm size, 40-nm-thick flakes (aspect ratio of ~1:500), and (f) a 380-nm size, 20-nm-thick flake (aspect ratio of approximately 1:20). To further verify that the produced nano- and microstructures are made of MoS_2_, we used the energy dispersive X-ray spectroscopy (EDS) module in the SEM system (Model: Tescan MIRA3 FEG SEM; acceleration voltage: 5 kV; dwell time: 200 μs) to acquire EDS spectra and corresponding elemental mapping images from the regions bearing the target nano- and microstructures. For example, Figure 3g displays the integral EDS spectrum obtained from the nanoscale flake shown in Figure 3f. This EDS spectrum clearly shows the peak associated with MoS_2_. In addition, Figure 3h shows the corresponding EDS elemental mapping image of Mo (Red) and Si (Green) in which the MoS_2_ feature exhibits an identifiable contrast against the Si background. This demonstrates that EDS can be used as a tool for identifying sub-micrometer MoS_2_ features. These results also indicate that the observed nano- and microstructures are indeed made of MoS_2_.

**Figure 4:**
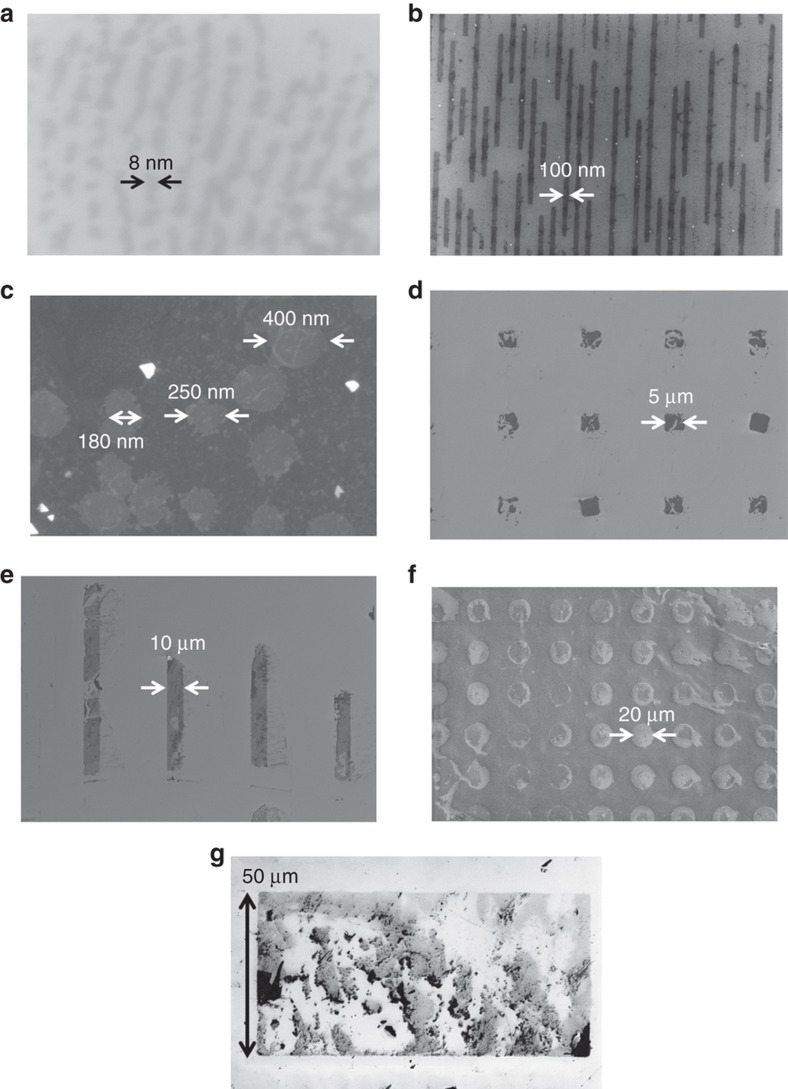
SEM images of representative bad quality MoS_2_ nano- and microstructures with feature aspect ratios (feature thickness/lateral size ratios) smaller than the critical aspect ratios, which results in major defects in the exfoliated structures: (**a**) 8 nm half-pitch, 0.6 nm-thick line-spacing patterns, (**b**) 100 nm half-pitch, 2 nm-thick line-spacing patterns, (**c**) hundreds of nm size, 3.5 nm-thick flakes, (**d**) 5 μm size, 8 nm-thick flakes, (**e**) 10 μm size, 20 nm-thick lines, (**f**) 20 μm size, 33 nm-thick flakes, (**g**) 50 μm wide, 70 nm-thick (average thickness) film.

Figure 4 displays the SEM images of the representative NASE+TP-produced MoS_2_ nano- and microstructures with relatively small aspect ratios, which exhibit major defects caused by shear exfoliation. These exfoliated MoS_2_ nano- and microstructures include (a) an 8 nm half-pitch, 0.6 nm-thick line-spacing structures (aspect ratio approximately 1:14), (b) a 100 nm half-pitch, 2 nm-thick line-spacing structures (aspect ratio approximately 1:50), (c) hundreds of nanometer-sized, 3.5 nm-thick mesas (aspect ratio approximately 1:29), (d) 5 μm size, 8 nm-thick flakes (aspect ratio approximately 1:625), (e) 10 μm size, 20 nm-thick lines (aspect ratio approximately 1:500), (f) 20 μm size, 33 nm-thick flakes (aspect ratio approximately 1:600), and (g) 50 μm wide, 70 nm-thick (average thickness) film (aspect ratio approximately 1:710).

## Discussion

**Figure 5:**
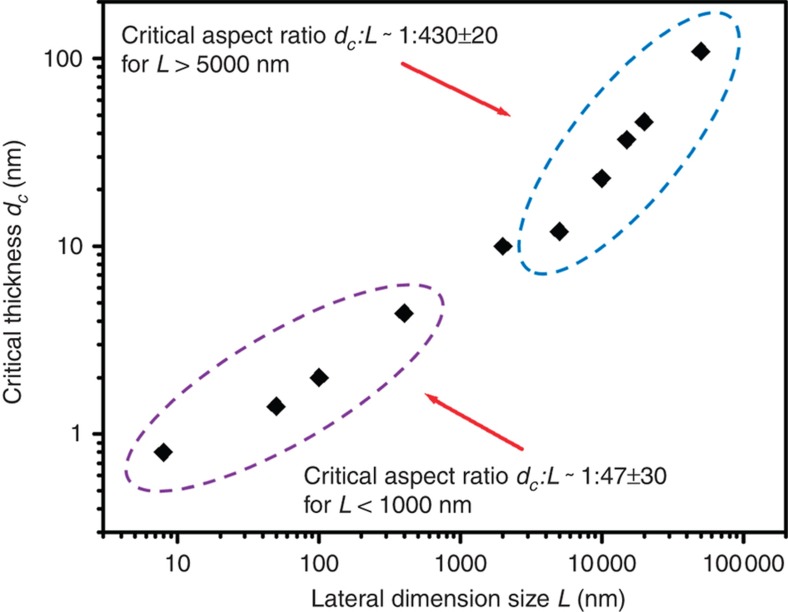
Critical thickness (*d*_c_) values plotted as a function of the corresponding characteristic lateral dimension sizes (*L*).

Our NASE+TP test results of the MoS_2_ nano- and microstructures with various lateral feature sizes and vertical thicknesses indicate that for a given characteristic lateral size of pre-patterned MoS_2_ a nano- or microstructure on the bulk MoS_2_ stamp, the vertical thickness (or aspect ratio) of these structures has a critical limit (*d*_c_); below this critical limit to-be-exfoliated MoS_2_ nano- and microstructures have a poor mechanical rigidity, and as-exfoliated nano/structures typically exhibit major defective features (that is, a very low yield of high-quality MoS_2_ structures that are free of mechanical damage) that are caused by the shear exfoliation. More specifically, Figure 5 plots the experimentally determined critical thickness (*d*_c_) values as a function of characteristic lateral feature sizes (*L*). As shown in Figure 5, for the exfoliated MoS_2_ structures with lateral feature sizes larger than 5 μm, the critical aspect ratio is estimated to be 1:430±20, whereas for the MoS_2_ nanostructures with lateral sizes smaller than 1 μm, the critical aspect ratio to assure a damage-free exfoliation is approximately 1:47±30. This result implies that to produce high-quality ultrathin monolayer or few-layer MoS_2_ structures using NASE+TP or other mechanical exfoliation processes, the lateral dimensions of the protrusive to-be-exfoliated MoS_2_ features on the bulk stamp need to be patterned in sub-100-nm regimes. The critical feature aspect ratio required for generating high-quality MoS_2_ structures increases with the reduction of the targeted feature thickness (or the targeted lateral size). Thus, in comparison with the production of relatively thick multilayer MoS_2_ structures (typically thicker than 10 nm), the production of ultrathin monolayer or few-layer MoS_2_ structures using NASE+TP processes needs a larger feature aspect ratio to assure that the MoS_2_ nanostructures have a sufficiently high rigidity and can survive shear exfoliation processes. This trend of requiring larger feature aspect ratios for high-yield exfoliation of monolayer/few-layer structures probably occurs because when the thickness of to-be-exfoliated MoS_2_ structures is pre-defined in monolayer/few-layer regimes (that is, typically, 0.5–7 nm), the nanoscale surface roughness of the MoS_2_ stamp and naturally formed crystal terraces with nanoscale step heights could mechanically interact with as-exfoliated MoS_2_ nanostructures. Because of the comparable dimension sizes between roughness features and exfoliated ultrathin MoS_2_ structures, such mechanical interaction easily leads to severe damage to the as-exfoliated MoS_2_ structures. Therefore, the thinner to-be-exfoliated structure typically needs a larger feature aspect ratio (that is, a higher structure rigidity) to survive in the exfoliation process. For the exfoliation of relatively thick multilayer structures (typically thicker than 7 nm), such an effect on the surface roughness features is not expected to be significant because of the large discrepancy between exfoliated MoS_2_ structures and surface roughness features regarding their dimension sizes. The most important physical significance of the critical thickness (or aspect ratio) data plotted in Figure 5 is that they provide a quantitative limit profile for the MoS_2_ feature dimensions enabled by NASE+TP, which is designed to generate MoS_2_ device structures with a high uniformity in feature size. Although the specific values of critical thicknesses or aspect ratios are expected to be different for various layered materials or various exfoliation methods, the basic scaling behaviors (or trends) of the critical aspect ratios for these different cases are still expected to be similar. This is because the aspect ratios of to-be-exfoliated layered nano- and microstructures always play a critical role in determining the rigidity of such features, which always significantly affects the yield of good-quality features generated by various mechanical exfoliation methods. Furthermore, the method presented here for measuring the critical aspect ratios of NASE+TP-produced MoS_2_ structures can serve as a generic methodology for determining the critical aspect ratios of various layered materials generated via different exfoliation methods.

**Figure 6:**
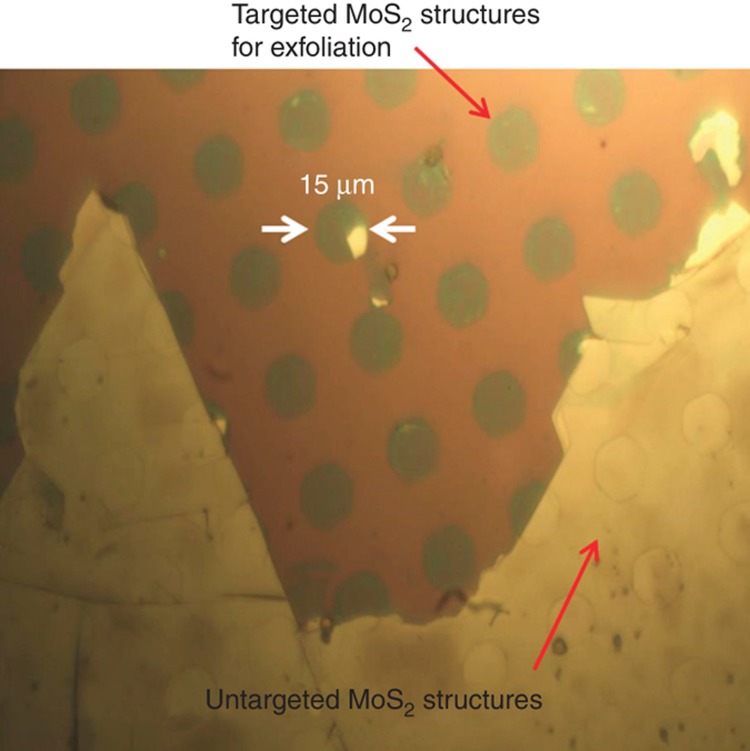
Optical micrograph of as-exfoliated 15 μm-sized, 20 nm-thick MoS_2_ mesas and untargeted MoS_2_ layers, which are exfoliated due to the poor exfoliation selectivity between the pre-patterned (or targeted) and unpatterned (or untargeted) MoS_2_ layers. This poor selectivity is usually observed when the aspect ratio of pre-patterned to-be-exfoliated MoS_2_ structures is close to or smaller than the critical value.

Another concomitant effect of the surface roughness and crystal terrace features is that when the aspect ratio (or thickness) of pre-patterned to-be-exfoliated MoS_2_ structures is close to or smaller than the corresponding critical value, the selectivity for preferred exfoliation of pre-patterned (or targeted) MoS_2_ layers over undesired exfoliation of unpatterned (or untargeted) MoS_2_ layers becomes significantly lower. For example, Figure 6 shows an optical micrograph of an array of as-exfoliated 15-μm size, 20-nm-thick MoS_2_ mesas (corresponding critical thickness, *d*_c_, ~30 nm) and the untargeted underlying MoS_2_ layers. Such untargeted layers are not lithographically patterned but are usually exfoliated due to the poor exfoliation selectivity between pre-patterned and unpatterned MoS_2_ layers. More specifically, in the case of exfoliation of relatively thick pre-patterned MoS_2_ structures (*d*»*d*_c_), the shear stress is dominantly accumulated in the pre-patterned MoS_2_ layers with relatively thick protrusive edges. However, in the case of the exfoliation of relatively thin structures (*d*<*d*_c_), since the surface roughness is comparable to the pre-patterned feature thickness (or height), a significant portion of the total shear force is exerted on the roughness or crystal terrace features in unpatterned MoS_2_ layers, resulting in a more even distribution of the shear stress between pre-patterned and unpatterned layers (or regions) and leading to a poor exfoliation selectivity.

**Figure 7:**
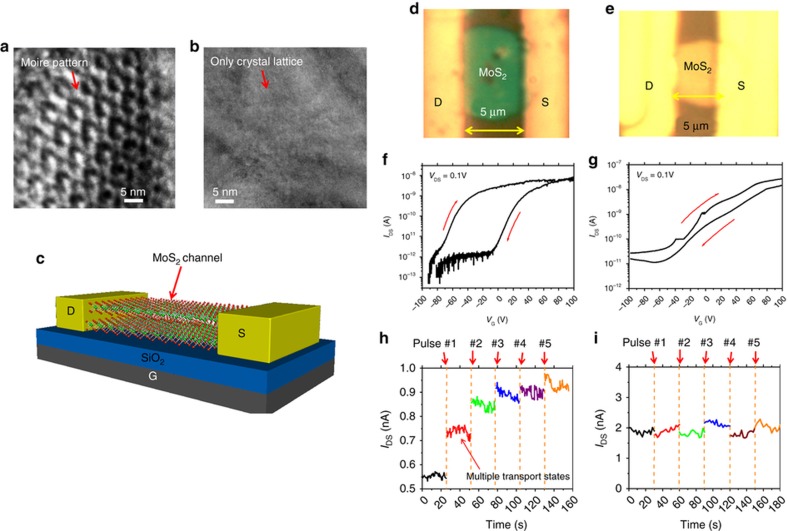
Charge memory characteristics of the FETs made from MoS_2_ flakes: (**a**) and (**b**) show the HRTEM images of the cleaved surfaces of one MoS_2_ flake of critical thickness (or critical aspect ratio) and the other flake, which is thicker than the critical thickness, respectively; (**c**) schematic illustration of a back-gated MoS_2_ FET; (**d** and **e**) show the optical micrographs of the FETs made from the MoS_2_ flakes shown in (**a** and **b**), respectively; (**f** and **g**) display *I*_DS_—*V*_G_ curves measured from these two FETs, respectively; (**h** and **i**) display the *I*_DS_—*t* curves measured from the FETs made from the MoS_2_ flakes shown in (**a** and **b**), respectively, which are modulated by a set of periodic −50 V, 100 ms *V*_G_ pulses.

We also obtained high-resolution transmission electron micrographs (HRTEMs) of the as-exfoliated MoS_2_ structures with feature thicknesses (*d*) close to the corresponding critical values (*d*_c_) and for those with *d»d*_c_. The exfoliated MoS_2_ structures with *d*~*d*_c_ have exfoliation-induced interlayer deformation features. For example, Figures 7a and b show the HRTEM images of the cleaved surfaces of one as-exfoliated MoS_2_ flake with a lateral size, *L*, of ~5 μm and a thickness, *d*, of ~8 nm (corresponding critical thickness, *d*_c_, of ~10 nm) and the other flake with a *L* of ~5 μm and a *d* of ~18 nm (*d*_c_ is still ~10 nm), respectively. The MoS_2_ flake with *d*~*d*_c_ exhibits a quasi-periodic Moiré pattern on its cleaved surface, which indicates the existence of exfoliation-induced interlayer twist features in the MoS_2_ layers close to the cleaved surface. The cleaved surface of the MoS_2_ flake with *d*>*d*_c_ does not exhibit any Moiré patterns but features a high-quality crystal lattice, indicating no exfoliation-induced interlayer deformation or twisting in the exfoliated layers.

The interlayer twisting angle (*θ*) and the resulting Moiré pattern period (*P*) in MoS_2_ layers can be evaluated using *P=a/θ* in which *a* is the in-plane lattice constant (for MoS_2_, *a*=0.318 nm). The Moiré pattern periods were observed in the MoS_2_ flake with the *d*~*d*_c_ range from 3 to 6 nm, from which the interlayer twisting angles were estimated to be 3–6°. As implied by several previous theoretical works, the Moiré patterns in few-layer TMDC structures could spatially modulate the interlayer coupling of electronic band states that originated from different layers, resulting in spatial trapping of charged carriers and spatial variation of electrostatic potential^16–18^. Such a Moiré pattern effect, if introduced into a field-effect transistor structure, is expected to result in charge memory states. To support this speculation, we further investigated and compared the charge-trapping characteristics of the FETs made from the MoS_2_ flakes in the cases of *d~d*_c_ and *d*>*d*_c_. Figure 7c schematically illustrates the back-gated MoS_2_ FET structure under study. For such an FET, the MoS_2_ channel length is ~4 μm, and the channel width is ~5 μm. A pair of Ti (5 nm)/Au (50 nm) electrodes were fabricated using photolithography followed by metallization to serve as source-drain (D–S) contacts. The p^+^–Si substrate serves as the back gate (G) for modulating the MoS_2_ channel. The thermally grown 300 nm-thick SiO_2_ layer on top of the Si substrate serves as the gate dielectric. The FETs were characterized using a LakeShore probe station (Lake Shore Cryotronics Inc., Westerville, OH) connected to a HP4145 semiconductor parameter analyzer. Figures 7d and e display the optical micrographs of two representative MoS_2_ FETs in the cases of *d*~*d*_c_ and *d*>*d*_c_, respectively (that is, the FETs made from the MoS_2_ flakes very similar to the ones shown in Figures 7a and b). Figures 7f and g show the drain-source current (*I*_DS_)—gate voltage (*V*_G_) curves (or transfer characteristic curves) of these two devices. The transfer curves in Figure 7g exhibit a couple of small kinks. These kinks should be attributed to the interfacial states at the MoS_2_/SiO_2_ interfaces. The charging or discharging events associated with these states usually result in small kink features on transfer characteristic curves. However, such charge-trapping states have very short retention times and do not prominently affect the charge retention characteristics of interlayer deformation-induced charge-trapping states in MoS_2_ layers. The On/Off current ratio of the FET with *d*~*d*_c_ (*I*_ON_*/I*_OFF_ ~10^4^) is slightly larger than that of the one with *d*>*d*_c_ (*I*_ON_*/I*_OFF_~10^3^). This is because the thinner MoS_2_ FET channel usually results in a higher effectiveness of the gate potential for modulating the channel conductance. However, for both FETs, their On/Off ratios are high enough to enable a dynamic range of the channel conductance (or *I*_DS_ measured under a given set of *V*_DS_ and *V*_G_ biases) for observing the *V*_G_-mediated charge-trapping processes in the FETs. Specifically, a queue of −50 V, 100-ms *V*_G_ pulses with pulse-to-pulse spacing of 30 s were sequentially applied to the FET under study. During the intervals between *V*_G_ pulses, the time-dependent *I*_DS_ values (that is, *I*_DS_*−t* curves) were measured under fixed *V*_DS_ and *V*_G_ (typically *V*_G_=0 and *V*_DS_=0.05 V) to show the *V*_G_-mediated charge-trapping processes in the FET. Figures 7h and i display the *I*_DS_−*t* curves measured from the MoS_2_ FETs in the cases of *d~d*_c_ and *d>d*_c_, respectively. We observed that the FET with *d*~*d*_c_ exhibits a cascading increase of *I*_DS_ in response to the time-sequenced *V*_G_ pulses, whereas the FET with *d*>*d*_c_ does not show a significant change in the *I*_DS_ level, and the slight fluctuation in *I*_DS_ is not relevant for the applied *V*_G_ pulses. This characterization result shows that the FET with *d~d*_c_ has *V*_G_-tunable charge-trapping states that do not exist in the FET with *d*>*d*_c_. In one of our recent works, we found that few-layer WSe_2_ FETs, once they bear interlayer deformation nanostructures, exhibit multiple long-lasting charge-trapping states^9^. Therefore, the *V*_G_-tunable charge-trapping states observed in the present work can also be reasonably attributed to the interlayer twisting features observed in the MoS_2_ channel with *d~d*_c_. This FET result is consistent with the TEM result mentioned above and further indicates that the NASE+TP-produced MoS_2_ structures with feature thickness close to *d*_c_ likely bear interlayer twisting or other deformation features, which can result in *V*_G_-tunable charge-trapping states in the FETs made from such MoS_2_ structures. Such charge-trapping states could be further studied for making multi-bit or quasi-analog-tunable memory devices for emerging neuromorphic and analog computing device applications. In addition, our MoS_2_ FETs with lengths/widths in the range of 2–20 μm, if their aspect ratios are close to the corresponding critical values, exhibit very similar charge-trapping characteristics. However, it is still not clear whether or not the MoS_2_ FETs with nanoscale lengths or widths have similar charge-trapping states due to interlayer deformation. To fully understand this question, we need methods for making nanoscale MoS_2_ FETs without populating the charging states associated with other sources. In particular, in our current fabrication method, the plasma etching processes always introduce additional charge-trapping states at the feature edges, which dominate the charging characteristics of nanoscale MoS_2_ FETs (note that such charging states at the feature edges are not dominant in microscale MoS_2_ FETs.) In future work, we will seek to rule out the effects of such charging states at the feature edges of nanoscale MoS_2_ FETs and therefore identify the interlayer deformation-mediated charge-trapping characteristics for these types of nanoscale devices.

## Conclusions

In summary, we studied the critical limit of the feature dimensions of MoS_2_ nano- and microstructures produced via NASE+TP processes. In particular, we explored the processing condition for producing ultrathin monolayer and few-layer MoS_2_ structures. Our work shows that the quality and yield of NASE+TP-produced MoS_2_ nano- and microstructures are dependent on the aspect ratio of pre-patterned to-be-exfoliated MoS_2_ structures on a bulk stamp. For the pre-patterned MoS_2_ structures with a given lateral feature size, the vertical thickness (or aspect ratio) of these structures has a critical limit; below this critical limit, the as-exfoliated MoS_2_ nano- and microstructures are easily damaged by the NASE processes. Our experiments specifically indicate that for MoS_2_ structures with lateral feature sizes of >5 μm, the critical aspect ratio is estimated to be approximately 1:430, whereas for MoS_2_ nanostructures with lateral sizes of <1 μm, the critical aspect ratio is approximately 1:47. This indicates that to generate ultrathin monolayer or few-layer MoS_2_ structures that are free of mechanical damage, the lateral dimensions of the protrusive MoS_2_ structures on the stamp need to be pre-patterned in the sub-100-nm regime. Furthermore, we found that the as-exfoliated MoS_2_ structures with thicknesses or aspect ratios very close to the corresponding critical values bear interlayer twisting features that result in top-view Moiré patterns on the cleaved surfaces. The FETs made from these MoS_2_ layers exhibit multiple *V*_G_-tunable charge memory states. This work advances the understanding of the limitation and application scope of nanoimprint and nanoprint processes for manufacturing MoS_2_ and other TMDC device structures. The identified charge memory states in the MoS_2_ FETs with thicknesses close to the critical values could be further studied and exploited to make analog computing and multi-bit memory devices.
